# *RAB27A* silencing modulates miR-30d-5p expression and extracellular vesicle non-coding RNA cargo in diabetic podocytes

**DOI:** 10.1038/s41598-026-49921-1

**Published:** 2026-04-24

**Authors:** Olga Martinez-Arroyo, Ana Flores-Chova, Lesley Escriva, Marta Mendez-Debaets, Sergio Martinez-Hervas, Cristina Grange, Benedetta Bussolati, Maria Jose Forner, Josep Redon, Raquel Cortes, Ana Ortega

**Affiliations:** 1 Cardiometabolic and Renal Risk Research Group, Biomedical Research Institute of Hospital Clinico de Valencia INCLIVA, Valencia, Spain; 2 Service of Endocrinology and Nutrition, Hospital Clinico Universitario of Valencia, 46010 Valencia, Spain; 3 Cardiometabolic Risk and Diabetes Group, Biomedical Research Institute of Hospital Clinico de Valencia INCLIVA, Valencia, Spain; 4 Department of Medicine, Faculty of Medicine, University of Valencia, 46010 Valencia, Spain; 5 CIBERDEM (CIBER of Diabetes and Associated Metabolic Diseases), 28029 Madrid, Spain; 6 Department of Medical Sciences, University of Turin, 10126 Turin, Italy; 7 Internal Medicine Unit, Hospital Clinico Universitario, 46010 Valencia, Spain; 8 CIBEROBN (CIBER of Pathophysiology of Obesity and Nutrition), 28029 Madrid, Spain; 9 CIBERCV (CIBER of Cardiovascular Diseases), 28029 Madrid, Spain

**Keywords:** Diabetic nephropathy, Podocytes, Rab27A, Non-coding RNAs, Extracellular vesicles, miR-30d-5p, Cell biology, Diseases, Molecular biology, Nephrology

## Abstract

Diabetic nephropathy (DN) is one of the leading causes of end-stage renal disease and is characterised by early podocyte injury and loss. Increasing evidence indicates that extracellular vesicles (EVs) are key mediators of intercellular communication in the diabetic kidney, carrying bioactive molecules such as non-coding RNAs (ncRNAs) that influence disease progression. However, the identity of ncRNAs selectively loaded into podocyte EVs under hyperglycaemia and the contribution of vesicle-trafficking regulators such as *RAB27A* to this process remain to be elucidated. In this study, conditionally immortalised human podocytes were used to model diabetic conditions through high glucose (HG) exposure. To explore the role of vesicular trafficking in ncRNA release, *RAB27A*, an essential regulator of EV secretion, was silenced using siRNA. Small RNA sequencing was performed to characterize the EV ncRNA profile and subsequent RT-qPCR was used to validate results. Our findings revealed that HG and *RAB27A* silencing significantly altered the EV ncRNA composition, with a marked downregulation of miR-30d-5p. Additional experiments revealed that miR-30d-5p influences its target *SMAD1*, involved in antifibrotic signalling pathways. Collectively, these results propose that *RAB27A*-dependent vesicle trafficking and glucose stress regulate the ncRNA landscape of podocyte-derived EVs, positioning miR-30d-5p as a mediator of SMAD1-related pathways during hyperglycaemic stress.

**Supplementary Information:**

The online version contains supplementary material available at 10.1038/s41598-026-49921-1.

## Introduction

Diabetic nephropathy (DN) is a leading cause of end-stage renal disease worldwide and is characterised by progressive glomerular damage, proteinuria, and eventual renal failure^1,2^. Podocytes, highly specialised epithelial cells forming part of the glomerular filtration barrier, are essential for maintaining the structural and functional integrity of the glomerulus. Their dysfunction and depletion are hallmark features of DN, yet the underlying molecular mechanisms remain incompletely understood^3,4^.

Recent research has highlighted the importance of intra and intercellular trafficking processes in podocyte biology, particularly in maintaining slit diaphragm structure and regulating vesicular communication^5–7^. Within this context, RAB27A, a member of the Rab family of small GTPases, plays a key role in the docking and exocytosis of vesicles across multiple cell types, including secretory and immune cells^8–10^. In podocytes, the precise functions of RAB27A remain underexplored. However, previous studies of our group, analysed the Rab–Rabphilin system under injurious stimuli and directly tested the consequences of interfering with Rab3A/Rab27A signalling in podocytes. Specifically, we reported alterations in the Rab–Rabphilin axis in human podocytes exposed to glucose overload and angiotensin II^11^ and subsequently showed that silencing the Rab3A/Rab27A system under glucose stress, ameliorates high glucose-induced injury in podocytes by reducing apoptosis, improving cytoskeletal integrity (less disruption), and normalizing the vesicle distribution of CD63-positive vesicles^12^. Although these findings indicate that Rab-dependent trafficking contributes to podocyte responses to metabolic stress, they do not explain which pathways could be affected by RAB27A silencing; therefore, defining RAB27A’s roles in podocytes and their potential impact on DN is of clear interest.

Extracellular vesicles (EVs), small, membrane-bound particles secreted by mainly all cell types and present in almost all biofluids, carry proteins, lipids and nucleic acids and mediate intercellular communication by transferring functional cargo between cells^13^. In the kidney, EVs are released from multiple renal cell types (including tubular cells, endothelial cells and podocytes) and are increasingly recognised both as mediators of local cell–cell signalling and as a source of urinary and plasma biomarkers for kidney injury^14–18^. Small non-coding RNAs (ncRNAs), as microRNAs (miRNAs) are highly enriched in EVs. miRNAs have been implicated in fibrotic signalling, apoptosis/survival and TGF-β pathways in renal damage associated diseases, including DN^19–21^, but the identity of specific ncRNAs packaged by podocyte EVs under hyperglycaemia and the role of vesicle-trafficking regulators such as RAB27A in this process remains to be established.

In this study, we aimed to investigate how RAB27A silencing in human podocytes exposed to hyperglycaemic conditions influences the molecular content of their secreted EVs, with a particular focus on small ncRNAs.

## Results

### EV characterization from podocyte cultures

**Fig. 1:**
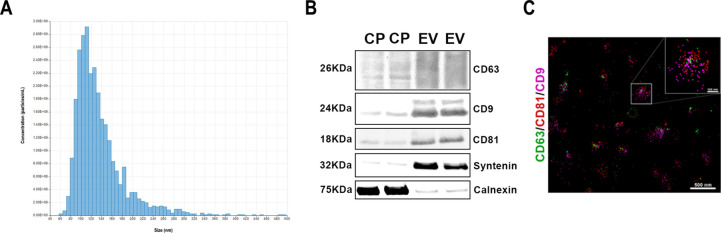
Characterisation of podocyte-derived EVs. (**A**) Tunable Resistive Pulse Sensing analysis showing the size distribution and concentration of EVs. (**B**) Cropped membranes from Western blot analysis of EV protein markers and organelle marker calnexin. EV fractions expressed the canonical tetraspanins CD63, CD9 and CD81, as well as the late endosomal protein syntenin. (**C**) Super-resolution dSTORM imaging of EVs labelled for CD63 (green), CD81 (red), and CD9 (magenta). Scale bar 500 nm. CP, cell pellet; EV, extracellular vesicle. Whole membranes from the Western blot are available in the supplementary material as (Fig. S2).

EVs released by cultured podocytes were characterised using complementary approaches. TRPS analysis revealed a heterogeneous distribution of particle sizes, with a peak centred around 90–120 nm, consistent with the expected size range of small EVs (Fig. 1A). In addition, we have quantified both size and quantity under all experimental conditions and as shown in Fig. S1, we did not observe differences that reached statistical significance in either particle concentration or size distribution between control and Rab27a-silenced podocytes. Further analysis in EVs by Western blotting confirmed the presence of the EV-associated markers CD63, CD9, CD81 and syntenin in EV fractions, while calnexin, was detected only in podocyte cell pellet lysates, indicating minimal contamination from intracellular organelles (Fig. 1B and Fig. S2). Super-resolution microscopy further validated the vesicular identity, showing distinct labelling of CD63, CD9 and CD81 at the single-vesicle level, with several EVs displaying co-localisation of multiple tetraspanins (Fig. 1C). Together, these findings confirm the successful isolation of pure populations of podocyte-derived EVs. To ensure transparent reporting in EV research, data regarding EV characterization has been deposited in EV-TRACK under the accession number EV260003.

### Global shifts in EV RNA biotypes after RAB27A knockdown and high glucose exposure

**Fig. 2:**
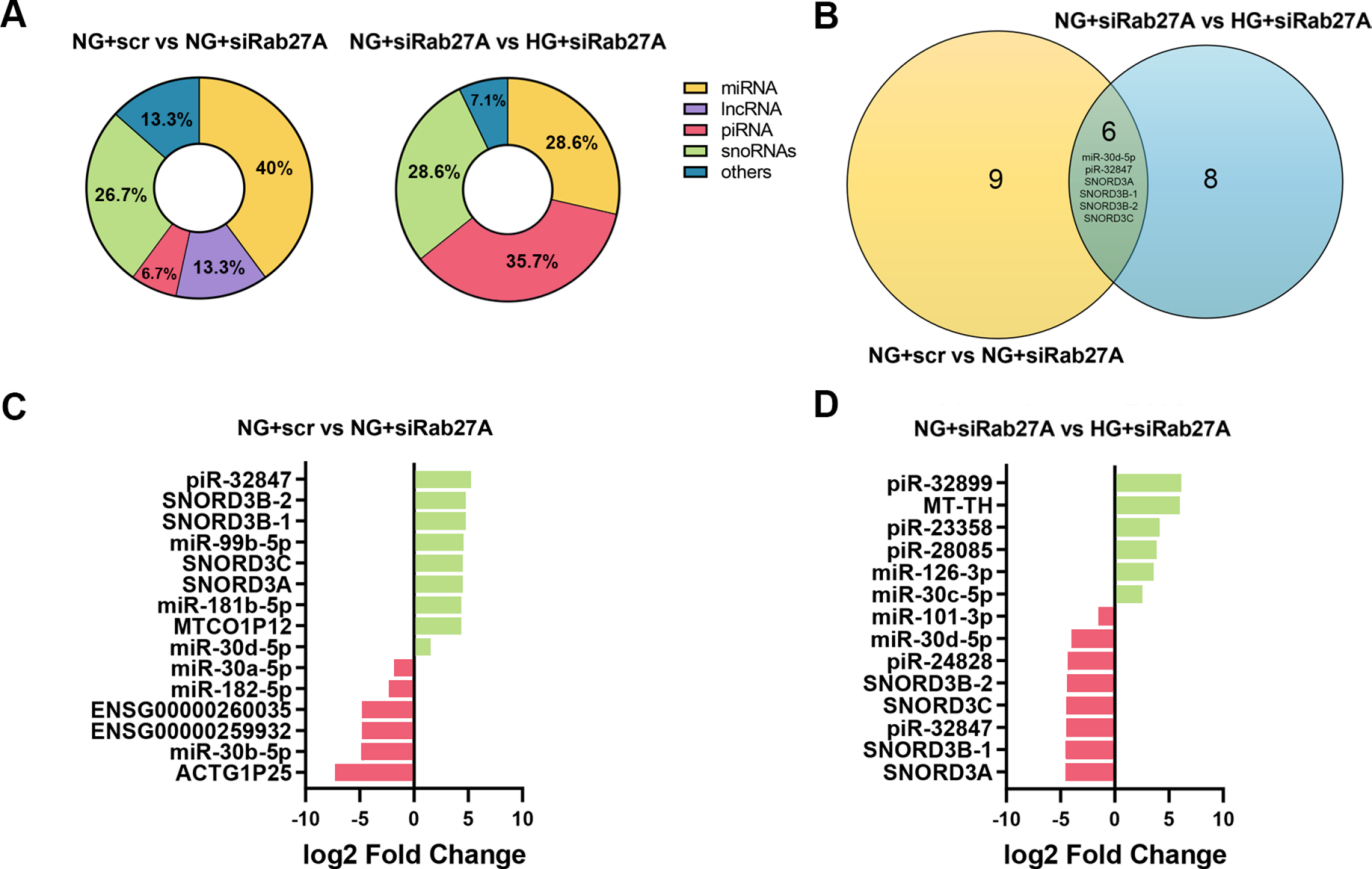
Differential small non-coding RNA (ncRNA) content of EVs from RAB27A-silenced podocytes under normal and high glucose treatments. (**A**) Distribution of differentially expressed (DE) small ncRNA biotypes across two pairwise comparisons: NG + scr vs NG + siRAB27A (left) and NG + siRAB27A vs HG + siRAB27A (right). Percentages represent the fraction of all DE ncRNAs assigned to each biotype (miRNA, lncRNA, piRNA, snoRNA, others (transcribed_processed_pseudogene, unprocessed_pseudogene, Mt_tRNA)). (**B**) Venn diagram showing the number of DE small ncRNA from NG + scr vs NG + siRAB27A (left circle, yellow) and NG + siRAB27A vs HG + siRAB27A (right circle, blue) and the common ncRNAs in both comparisons (green). (**C**) Top DE small ncRNAs for NG + scr vs NG + siRAB27A. (**D**) Top DE ncRNAs found in NG + siRAB27A vs HG + siRAB27A. Bars to the right of the vertical line indicate upregulated ncRNAs (green); bars to the left indicate downregulated ncRNAs (red). ncRNA identifiers are shown on the y-axes.

RAB27A silencing produced widespread changes in the small ncRNA content of podocyte-derived EVs, and these changes were further modulated by HG (Fig. 2). In the NG + scr vs NG + siRAB27A comparison, most differentially expressed (DE) ncRNAs were miRNAs and snoRNAs, whereas the NG + siRAB27A vs HG + siRAB27A comparison showed that miRNAs, piRNAs and snoRNAs were the most DE ncRNAs, alongside a reduced contribution from lncRNAs, suggesting that hyperglycaemia may alter the impact of RAB27A knockdown on EV RNA composition (Fig. 2A). A Venn diagram analysis (Fig. 2B), revealed that six ncRNAs were commonly dysregulated in both comparisons Among them, only one miRNA was DE (miR-30d-5p), one piRNA (piR-32847) and 4 snoRNAs (SNORD3A, SNORD3B-1, SNORD3B-2 and SNORD3C). These shared changes point to a core set of ncRNAs that may be directly linked to Rab27A function. In contrast, additional subsets of ncRNAs were uniquely affected in each condition, indicating that glucose availability also modulates the ncRNA response to Rab27A knockdown. The directionality of these changes revealed distinct patterns depending on glucose treatment (Fig. 2C,D). In NG + scr vs NG + siRab27A (Fig. 2C), six ncRNAs were markedly downregulated, including miR-30a-5p, miR-128-5p, and SNORD3A, whereas nine, such as piR-32847, SNORD3B-2 and miR-30d-5p were upregulated. A distinct but partially overlapping profile was observed when comparing the effect of HG in silenced podocytes (Fig. 2D), where miRNAs (e.g. miR-30d-5p, mir-101-3p) and snoRNAs exhibited significant downregulation, contributing to a greater general downward trend in this comparison, with eight downregulated ncRNAs and six upregulated.

### miR-30d-5p analysis in podocytes

**Fig. 3:**
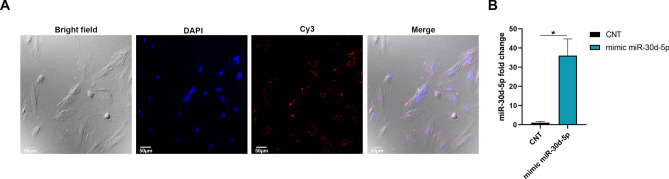
Validation of miR-30d-5p transfection efficiency in cultured podocytes. (**A**) Representative images of podocytes transfected with Cy3-labelled control oligonucleotides, showing bright field, DAPI staining of cell nuclei, Cy3 fluorescence, and merged channels, confirming successful uptake of transfected material. (**B**) RT-qPCR analysis demonstrated a robust increase in intracellular miR-30d-5p levels following transfection with a miR-30d-5p mimic compared to control group (CNT), confirming transfection efficiency. Data are shown as fold change relative to controls. Statistical significance is indicated as *p < 0.05.

To further explore the potential role of miR-30d-5p in vitro, we performed gain-of-function experiments using a synthetic mimic. As a first step, transfection efficiency was confirmed through the uptake of Cy3-labelled oligonucleotides, which showed robust intracellular fluorescence (Fig. 3A), and by RT-qPCR analysis, which demonstrated a strong increase in miR-30d-5p expression compared to control cells (Fig. 3B). These results validated the experimental approach and ensured that subsequent observations could be attributed to miR-30d-5p overexpression.

**Fig. 4:**
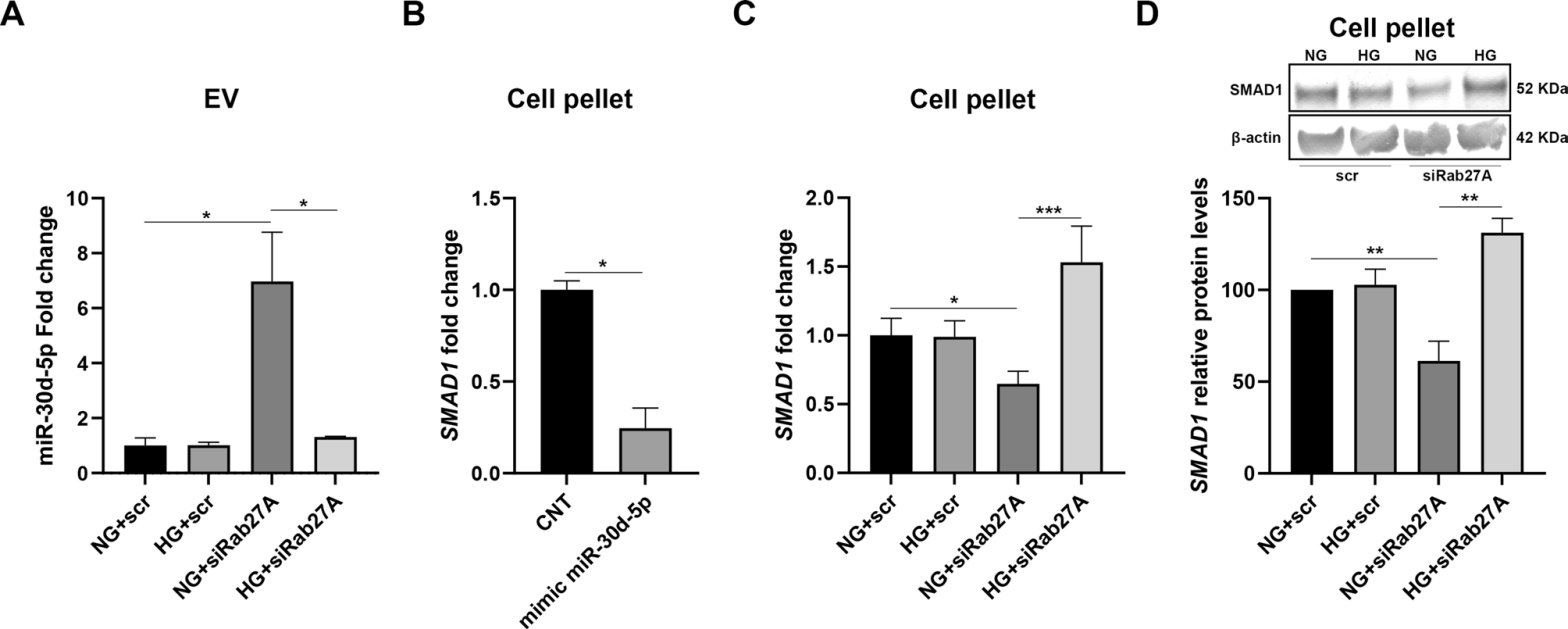
Effect of Rab27A silencing on miR-30d-5p and *SMAD1* expression in podocytes. (**A**) Relative expression of miR-30d-5p in EVs from podocytes cultured under normal glucose (NG) or high glucose (HG) conditions, with scrambled control (scr) or siRab27A transfection. (**B**) *SMAD1* expression in podocytes transfected with control (CNT) or miR-30d-5p mimic. (**C**) *SMAD1* expression in podocytes cultured under NG or HG conditions, with scr or siRab27A transfection. (**D**) SMAD1 protein levels in podocytes cultured under NG or HG conditions, with scr or siRab27A transfection. For the NG + scr or CNT group, mRNA levels are normalised to 1 and expressed as Fold change, and protein levels are normalised to 100. Fold change values were calculated relative to the NG scr or CNT conditions and normalised to appropriate endogenous controls (n = 5). Whole membranes from the Western blot are available in the supplementary material as (Fig. S3). Statistical significance is indicated as *p < 0.05, **p < 0.01, ***p < 0.001.

RT-qPCR validation of EV miR-30d-5p levels corroborated the sequencing results: compared with NG + scr, RAB27A knockdown in podocytes cultured in normal glucose produced a marked enrichment of miR-30d-5p in EVs, whereas HG + scr did not appreciably alter miR-30d-5p abundance; importantly, the increase observed after RAB27A silencing was largely abrogated when knockdown cells were maintained in HG (HG + siRAB27A) (Fig. 4A).

Given previous reports linking miR-30d-5p to the regulation of profibrotic and stress-related pathways, we next investigated whether its upregulation could influence known target genes in podocytes. In particular, SMAD1, a predicted and experimentally supported target of miR-30d-5p, was selected for further analysis.

Furthermore, forced overexpression of miR-30d-5p produced a significant reduction in *SMAD1* mRNA relative to control transfection (Fig. 4B), supporting that *SMAD1* is a miR-30d-5p target in these cells. Finally, analysis of *SMAD1* across glucose and Rab27A silencing conditions showed that *SMAD1* transcript levels were modulated by Rab27A knockdown and glucose stress (Fig. 4C). SMAD1 protein expression levels mirrored the trend observed at the mRNA level. Specifically, silencing of Rab27A resulted in a clear reduction in its protein abundance compared to control conditions. In contrast, when Rab27A-silenced podocytes were exposed to glucose treatment, protein levels increased relative to the silenced condition, indicating a partial recovery consistent with the transcriptional data (Fig. 4D and Fig. S4). These findings confirm that the regulation observed at the mRNA level is translated into corresponding changes in protein expression.

## Discussion

Podocyte injury is a key event in the onset and progression of glomerular damage in DN. As key components of the glomerular filtration barrier, podocytes rely on finely tuned intra- and intercellular communication to preserve glomerular homeostasis. EVs are critical mediators of this communication, by transferring bioactive molecules such as proteins, lipids, and nucleic acids between renal cells^22^. In this framework, small GTPases of the Rab family, including RAB27A, are crucial for vesicular trafficking and exocytosis, and their dysregulation may profoundly influence podocyte function and contribute to disease pathology^23,24^. Building on our previous work on Rab-Rabphilin system in podocytes, this study examines how RAB27A silencing in human podocytes exposed to hyperglycaemic conditions alters the ncRNAs composition of their secreted EVs.

Firstly, EVs characterisation confirmed that vesicles isolated from cultured podocytes fulfils standard criteria for small EVs showing a 90–120 nm size range, enrichment for canonical tetraspanins (CD63, CD9, CD81) and syntenin, and minimal contamination by the endoplasmic reticulum marker calnexin. These quality-control results provide a robust foundation for interpreting downstream RNA cargo analyses and align with current EV research recommendations^13^.

Regarding the role of Rab27a in the sorting of the vesicle cargo, reviews of Rab27 effectors emphasise that Rab27 proteins interact with multiple effectors to guide secretory vesicles to specific membrane fusion sites, supporting their role in selective cargo sorting defects following Rab27A knockdown^8^. Our transcriptomic analysis demonstrates that RAB27A depletion in human podocytes markedly reshapes the small ncRNA cargo of secreted EVs, including significant changes in miRNA profiles such as miR-30d-5p. Previous studies similarly link RAB27A to both EV secretion and cargo composition. For instance, Guo et al. demonstrated that RAB27A knockdown in melanoma cells leads to profound changes in exosome proteomic composition modulating metastatic potential of cancer cells^25^. Focusing in a renal environment, Liu et al. reported that RAB27A governs the release of albumin-containing exosomes in renal epithelial cells, suggesting a selective role in the trafficking or loading of specific protein cargos^26^. Wang et al. showed that RAB27A-dependent exosome secretion controls the export of miR-127-3p in renal carcinoma cells, linking RAB27A activity to selective miRNA packaging^27^. Together, our data reveal that RAB27A depletion in human podocytes alters the small ncRNA landscape of EVs, reinforcing the notion that RAB27A is a key regulator of both the quantity and qualitative composition of EV cargoes.

Among the small ncRNAs affected by RAB27A depletion, miR-30d-5p was the only miRNA consistently altered across the groups of interest. Interestingly, this effect was modulated by glucose stress: RAB27A knockdown increased EV miR-30d-5p under NG but diminished or reversed it under HG. Such modulation aligns with prior reports that hyperglycaemia remodels podocyte EV biology. For example, EVs from HG-treated podocytes carry distinct miRNA signatures that induce tubular dedifferentiation and injury, indicating that metabolic cues reshape EV cargo with functional implications across the nephron^17^. Similarly, studies showing miR-221-mediated tubular dedifferentiation driven by podocyte EVs in DN highlight that EV miRNA composition is both pathogenic and context-dependent^28^. These observations support a model in which metabolic stressors such as hyperglycaemia alter the miRNA-sorting machinery or trafficking pathways dependent on RAB27A, thereby reprogramming EV-mediated intercellular signalling within the glomerulus. In line with this, evidence from non-renal systems similarly indicates that RAB27A governs selective miRNA secretion^27^, suggesting that RAB27A-dependent mechanisms of miRNA loading and release are conserved across cell types.

Recent studies show that inhibiting miR-30d-5p enhances mitochondrial autophagy and mitigates HG-induced podocyte injury, indicating its maladaptive role under metabolic stress^29,30^. Consistently, our previous work demonstrated that RAB27A silencing under hyperglycaemia protects podocytes by restoring synaptopodin and nephrin levels, reducing apoptosis, and normalizing CD63 distribution^12^. Taken together, these findings raise the possibility that the beneficial effects of RAB27A depletion may partly result from reduced secretion of miR-30d-5p-enriched EVs, thereby limiting the pathogenic intercellular signalling. Overall, our data support a model in which RAB27A regulates both the quantity and quality of podocyte EVs, influencing the intercellular transmission of stress-related miRNAs such as miR-30d-5p and preserving glomerular homeostasis under hyperglycaemic conditions.

Given that miR-30d-5p was the most consistently altered EV miRNA and its modulation paralleled protective effects under RAB27A silencing, we investigated potential downstream targets. As shown by the miRNA target prediction databases and by prior studies, members of the miR-30 family, including miR-30d-5p, can target SMAD1^31,32^. Wu et al. demonstrated that miR-30 species bind the 3′ UTR of Smad1 and reduce its functional expression^33^. Notably, miR-30d-5p has been linked to podocyte protection, and its downregulation correlates with injury, supporting the biological relevance of the miR-30d-5p–SMAD1 axis in podocyte homeostasis^29,30^. Since BMP-7–SMAD1/5/8 signalling promotes renal repair and opposes profibrotic TGF-β/SMAD2/3 pathways, alleviation of miR-30d-5p–mediated repression may enhance protective BMP-SMAD signalling in the kidney^34–36^.

Interestingly, this interpretation aligns with our data: under glucose stress, RAB27A silencing reduces miR-30d-5p and preserves or increases SMAD1 expression, consistent with enhanced BMP–SMAD1–mediated protective signalling and our prior finding that RAB27A knockdown mitigates podocyte injury under glucose stress^12^. Another recent work by Hong et al*.* demonstrated that LRG1 promotes diabetic kidney disease progression by enhancing TGF-β–induced angiogenesis through ALK1–SMAD1/5/8 activation in glomerular endothelial cells^37^. In that model, excessive activation of the SMAD1/5/8 axis was associated with endothelial dysfunction, glomerular hypertrophy, and podocyte loss, whereas LRG1 ablation restores renal function. These findings the dual, context-dependent nature of SMAD1 signalling in the kidney. While excessive SMAD1 activation in endothelial cells may contribute to maladaptive angiogenesis and glomerular injury, controlled SMAD1 activity in podocytes might support cytoskeletal integrity and antifibrotic responses. In line with this interpretation, our results showing decreased miR-30d-5p and concomitant upregulation of SMAD1 upon RAB27A silencing under HG stress suggest that the restoration of SMAD1 signalling could represent an adaptive, protective mechanism mitigating podocyte injury in this context.

This study presents several limitations. Transient transfection restricts the timeframe for assessing silencing effects, limiting experimental observations duration. Moreover, although the experiments were carried out in an immortalised human podocyte line that reproduces key features of DN, this in vitro model cannot fully capture the complexity of the in vivo environment. In addition, RAB27A silencing, involved in EV secretion, inevitably reduced the vesicle yield available for experimental use, constraining downstream analyses. Future validation through podocyte-specific Rab27a silencing in animal models will be crucial to strengthen the translational relevance of these findings. Despite these limitations, this work provides a valuable framework for future investigations aimed at understanding the role of Rab27A silencing and its influence on vesicular trafficking in DN.

**Fig. 5:**
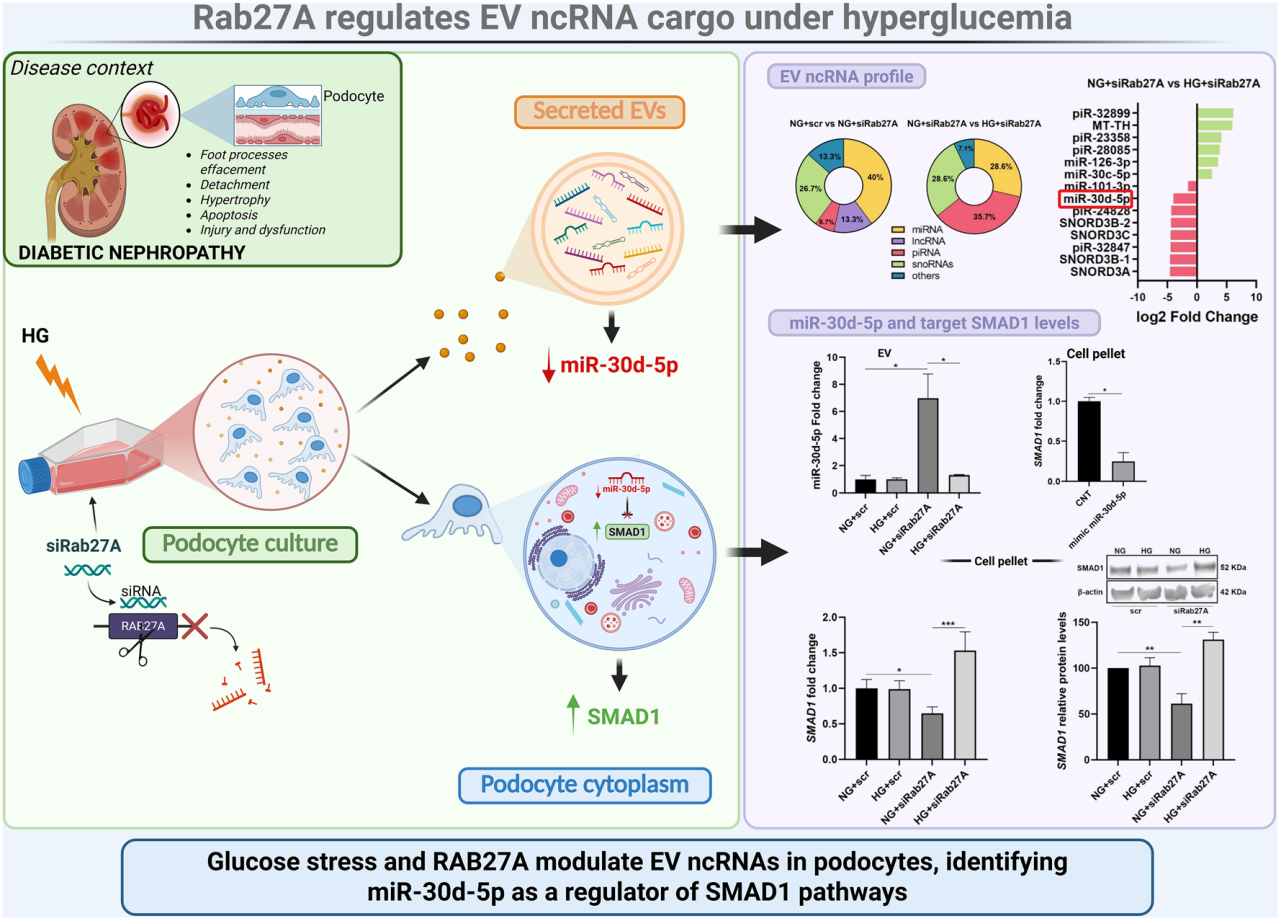
Schematic representation of Rab27A silencing and HG exposure on EV ncRNA profile. The results show the altered ncRNAs under silencing and treatment with glucose and reveal miR-30d-5p as a downregulated miRNA. Further analysis show how one target of miR30d-5p, SMAD1, is altered under silencing and HG at both mRNA and protein levels. CNT, control; EV, extracellular vesicle; HG, high glucose; ncRNA, non-coding RNA; NG, normal glucose; scr, scramble; siRNA, small interfering RNA; siRab27A, siRNA of Rab27A. Created with BioRender.com

In summary, the present study suggests that both RAB27A and glucose stress can be involved in the regulation of the ncRNA composition of podocyte‑derived EVs and identifies miR‑30d‑5p as a candidate mediator interacting with SMAD1 in the context of glucose stress (Fig. 5). The dependence of this sorting behaviour on metabolic state, highlights an underappreciated nexus between intracellular trafficking and extracellular communication in DN. Addressing the mechanistic details and in vivo consequences of Rab27A‑dependent EV secretion will be important next steps to evaluate both biomarker potential and therapeutic tractability in DN.

## Material and methods

### Podocyte cell culture

Conditionally immortalised human podocytes (cell line AB8/13), kindly provided by Prof. Moin Saleem (Children’s Renal Unit and Academic Renal Unit, University of Bristol, Southmead Hospital, Bristol, UK), were used for the experiments. At 33 °C, the SV40 T antigen is active, allowing cell proliferation. Upon shifting to 37 °C, the transgene is inactivated, leading to growth arrest and cellular differentiation^38^.

Cells were cultured on collagen type I-coated (Gibco, Thermo-Fisher Scientific, Waltham, MA, USA) flasks or plates in the Roswell Park Memorial Institute (RPMI) medium 1640 supplemented with 10% fetal bovine serum (FBS), 1% penicillin-streptomycin (both from Biowest, Nuaillé, France), and 1% insulin-transferrin-selenium (ITS, Gibco, Thermo-Fisher Scientific, Waltham, MA, USA). They were maintained at 33 °C until reaching 80% confluence, then trypsinised and reseeded at 70% confluence for differentiation that was induced by incubating cells at 37 °C with 2% FBS for 10–14 days. All cultures were kept in a humidified incubator with 5% CO₂ and 95% air (BINDER, VWR International, Radnor, PA, USA). Differentiated podocytes were used to perform all experiments.

### Transfection of siRNAs and glucose treatment

As reported previously by our group, podocytes were transiently transfected with siRNA targeting the RAB27A gene using Lipofectamine 3000 (Invitrogen, Thermo Fisher Scientific, Waltham, MA, USA) in serum-free Opti-MEM® Gibco, Thermo-Fisher Scientific, Waltham, MA, USA) according to the manufacturer’s protocol^12^. A pre-designed siRNA specific for RAB27A (Silencer™ Select, ID: s11693, Ambion, Invitrogen, Thermo Fisher Scientific, Waltham, MA, USA) was used. Transfection efficiency was monitored using a Cy3™ Dye-Labeled Negative Control siRNA (Invitrogen, Thermo Fisher Scientific, Waltham, MA, USA). As controls, cells were transfected with a scrambled siRNA (scr) (Silencer™ Select Negative Control, Invitrogen, Thermo Fisher Scientific, Waltham, MA, USA) and a GAPDH-targeting siRNA as a positive control (Silencer™ Select GAPDH siRNA, ID: 4404024, Invitrogen, Thermo Fisher Scientific, Waltham, MA, USA). Transient Rab27a silencing was verified at both the mRNA and protein levels and is available in reference^12^ as well as in the supplementary material (Figs. S4, S5).

After 24 h, the transfection medium was replaced with RPMI containing 1% penicillin–streptomycin and ITS. Cells were then treated with either normal glucose (NG; 5.5 mM) or high glucose (HG; 30 mM) concentrations (D-( +)-Glucose; Sigma Aldrich, St. Louis, MO, USA) and incubated for an additional 24 h at 37 °C.

### Transfection of synthetic miRNA mimics

Podocytes were transfected with miR-30d-5p mimic (hsa-miR-30d-5p Ambion™ mirVana™ miRNA Mimic ID: MC10756 ; Thermo Fisher Scientific, Waltham, MA, using Lipofectamine 3000 (Invitrogen, Thermo Fisher Scientific, Waltham, MA, USA) and Opti-MEM® (Gibco, Thermo Fisher Scientific, Waltham, MA, USA), the appropriate negative controls for mimic transfections (Ambion™ mirVana™ miRNA mimic negative control #1, ID: 4464058 from Thermo Fisher Scientific, Waltham, MA, USA) and culture medium without antibiotics, following the manufacturer’s instructions. After 24 h, transfection medium was replaced and treatments with glucose were performed as described above, and cells were incubated for 24 h at 37 °C.

### Size exclusion chromatography

Exosome isolation was performed using IZON size exclusion chromatography columns (qEVoriginal, Izon Science, Lyon, France). First, cell culture medium was recovered and centrifuged at 2,250 g for 30 min to remove apoptotic bodies and cell debris. Supernatant was then concentrated at 4,000 g for 15 min using 100 kDa cut-off Amicon Ultra 15 de 50 mL tubes (Millipore, Burlington, Massachusetts, USA). Once concentrated, samples were passed through the columns previously calibrated with phosphate-buffered saline (PBS) and fractions from 6 to 11 were recovered and concentrated again employing Amicon Ultra-4 Centrifugal Filter 10 kDa (Millipore, Burlington, Massachusetts, USA) at 4,000 g for 20 min. Isolated EVs were stored at −80 °C for further analysis.

### Tunable resistive pulse sensing (TRPS)

EVs derived from cell culture supernatants were analysed using an Exoid platform based in tunable resistive pulse sensing (TRPS) (Izon Science Ltd., Lyon, France). Prior to sample analysis, the nanopore (NP250) was calibrated with Izon calibration particles (TKP200, mean diameter 215 nm) to establish baseline parameters for size and concentration. EV suspensions were diluted in 0.22 µm filtered PBS and introduced into the measurement cell. Translocation of individual EVs through the nanopore was driven by carefully adjusted combinations of pressure, applied voltage, and nanopore stretch, optimised to achieve stable event rates within the target detection window. Calibration bead measurements were performed before and after EV runs to ensure accuracy and reproducibility. Each sample was run in triplicate. Data quality was continuously assessed through monitoring of baseline current stability, noise levels, and consistency of blockade profiles. Data were recorded and analysed using Izon Control Suite software v3.4 (Izon Science Ltd., Lyon, France; https://support.izon.com/how-can-i-get-the-latest-software-release).

### Homogenization of samples, gel electrophoresis and western blotting

Podocyte pellets and derived EVs were lysed in RIPA buffer (Thermo Fisher Scientific, Waltham, MA, USA) supplemented with a protease inhibitor cocktail (Sigma-Aldrich, St. Louis, MO, USA). Supernatants were centrifuged at 17,000 × g for 10 min at 4 °C, and the resulting supernatant was collected. Protein concentrations were determined using the Lowry method with bovine serum albumin (BSA) as the standard.

Proteins were separated on NuPAGE 4–12% polyacrylamide gels (Invitrogen, Carlsbad, CA, USA) and transferred onto polyvinylidene difluoride (PVDF) membranes. Membranes were blocked overnight and subsequently incubated with primary antibodies for 2 h at room temperature. For Rab27A and SMAD1 determination, mouse monoclonal anti-Rab27A (1:500, Abcam) and rabbit polyclonal anti-SMAD1 (1:3,000, Proteintech) were used and mouse monoclonal anti-β-actin antibody (1:6,000, Sigma-Aldrich) was used as a loading control. To characterize EV fractions, membranes were probed with the primary antibodies: rabbit monoclonal anti-CD9 (1:1000, Abcam), mouse monoclonal anti-CD63 (1:200, Abcam), mouse monoclonal anti-CD81 (1:250, Abcam), mouse monoclonal anti-syntenin (1:500, OriGene), rabbit monoclonal anti-calnexin (1:1000, Abcam).

After washing with Tris-buffered saline containing 0.1% Tween 20 (TBS-T; 20 mM Tris–HCl, 150 mM NaCl), membranes were incubated for 1 h at room temperature with alkaline phosphatase-conjugated anti-rabbit IgG or anti-mouse IgG secondary antibodies (Sigma-Aldrich, St. Louis, MO, USA). Membranes were then washed three times with TBS-T followed by TBS, and signal development was performed using 5-bromo-4-chloro-3-indolyl phosphate/nitro blue tetrazolium (BCIP/NBT; Sigma-Aldrich). Finally, band intensities were digitized using ImageQuant™ 7.0 TL software (https://info.cytivalifesciences.com/image-analysis-software.html).

### Super‐resolution microscopy

To investigate the expression of tetraspanins on the surface of EVs derived from podocytes, Super-Resolution Microscopy analysis was performed. Images were acquired with Nanoimager S Mark II system (Oxford Nanoimaging (ONI), Oxford, UK) equipped with a 100 × , 1.4NA oil immersion objective, an XYZ closed‐loop piezo 736 stage, and dual or triple emission channels split at 640 and 555 nm. Sample preparation was carried out according to previously described protocols^39,40^. EVs were stained with antibody reagents provided in the EV Profiler Kit (ONI, Oxford, UK). In brief, 7 µL of EV suspension were combined with 2 µL of blocking buffer (PBS supplemented with 5% BSA) and 1 µL of the antibody cocktail (anti-CD9-Atto488, anti-CD63-Alexa555, and anti-CD81-Alexa647) and incubated overnight at 4 °C.

On the following day, the mixture was deposited onto a pre-coated well and incubated for 1 h to allow vesicle attachment. Then, wells were washed twice with PBS and fixed with a fixative present in the Kit, before adding ONI B-Cubed Imaging Buffer (ONI, Oxford, UK). Super-resolution images were acquired in dSTORM mode using total internal reflection fluorescence (TIRF). Single-molecule datasets were filtered with NimOS software (v1.18.3, ONI; https://help.oni.bio/portal/en/kb/articles/nimos-overview), further processed with the Collaborative Discovery (CODI) online analysis platform (www.alto.codi.bio, ONI), and corrected for drift using version 0.2.3 of the correction algorithm^40^.

### RNA extraction

Total RNA, including small RNA fractions, was isolated from podocyte pellets using the miRNeasy Mini Kit (Qiagen, Hilden, Germany) and from podocyte-derived-EVs using the Total Exosome RNA and Protein Isolation kit (Invitrogen, Thermo Fisher Scientific, Waltham, MA, USA), following the manufacturer’s protocol. RNA yield and purity were quantified with a NanoDrop 2000 spectrophotometer (Thermo Fisher Scientific, Waltham, MA, USA). All RNA preparations were stored at − 80 °C until further use. For mRNA expression analysis, reverse transcription was performed using the Ready-To-Go You-Prime First-Strand Beads kit (GE Healthcare, Buckinghamshire, UK) according to the supplied instructions. For miRNA profiling, cDNA synthesis was carried out from 2 µL of total RNA employing the TaqMan™ Advanced miRNA cDNA Synthesis Kit (Applied Biosystems, Foster City, CA, USA). Resulting cDNA samples were preserved at − 80 °C until subsequent analyses.

### Small RNA library preparation, and next-generation sequencing

Libraries were prepared from 6 μL of total RNA per sample using the Small RNA-Seq Library Prep Kit (Lexogen GmbH, Vienna, Austria), following a protocol optimised for low-input samples^41^. After adapter ligation and cDNA amplification, libraries were size-selected (122–200 bp) on a 3% agarose gel using the Pippin Prep Automated DNA Size Selection system (Sage Science, Beverly, MA, USA), purified, concentrated, and quantified by Quantitative real-time polymerase chain reaction (RT-qPCR). Library quality was assessed by capillary electrophoresis on the QIAxcel Advanced System (Qiagen, Hilden, Germany). Individual libraries were normalised to 4 nM, pooled, and sequenced on the HiSeq X™ Ten platform (Illumina, San Diego, CA, USA) using 150-cycle paired-end reads.

Small RNA and RNA expression levels were quantified with the featureCounts function from the Rsubread package (Bioconductor, R;^42^) after trimming with Trim Galore! (https://www.bioinformatics.babraham.ac.uk/projects/trim_galore/) and alignment to the GRCh38 reference genome with STAR^43^. Annotation was performed using the GENCODE database for coding and ncRNAs, and miRBase, piRNAbank, and Ensembl for miRNAs and other small RNA species (snRNA, snoRNA, rRNA, piRNA).

RNA species with a base mean > 5 and detected in ≥ 3 samples with at least 1 read were retained. Differential expression analyses were performed with DESeq2^44^. P-values were adjusted using the Benjamini–Hochberg procedure, with FDR < 0.05 considered statistically significant. RNA-sequencing data generated in this study have been deposited in the Gene Expression Omnibus (GEO) under accession number **GSE310639**. The dataset contains raw and processed sequencing files obtained from EV RNA isolated from cell culture supernatant, together with the associated metadata. Data were prepared and submitted following GEO requirements to ensure transparency and reproducibility. The deposited files can be accessed freely for secondary analysis and validation purposes through the GEO repository.

### Quantitative real-time polymerase chain reaction (RT-qPCR)

Quantitative real-time polymerase chain reaction (RT-qPCR) was carried out on a LightCycler® 480 II system (Roche, Mannheim, Germany). Primers for mRNA analyses were designed with Primer3. For each assay, the appropriate primer pair was combined with Qiagen Multiplex PCR Master Mix and LC Green reagent (Qiagen, Hilden, Germany) and 2 µL of diluted cDNA was added to the reaction mixture.

For miRNA validation and overexpression analyses in cellular pellets and exosomes derived from podocyes, a TaqMan™ Small RNA Assay Protocol was followed. Briefly, a mix containing 1.33 μL of diluted cDNA, TaqMan™ Universal Master Mix II (no UNG), and specific TaqMan™ microRNA assay probe for miR-30d-5p (ID: 000420; Applied Biosystems, Foster City, CA, USA) was used.

All reactions were performed in triplicate and included suitable positive and negative controls, including a non-template control. Amplicon specificity was confirmed by melting-curve analysis. β-Actin (ACTB) and β2-microglobulin (B2MG) were used as endogenous reference genes for mRNA normalization, and cel-miR-39-3p (ID: 000200) served as an external spike-in control for miRNA assays. Relative expression levels were calculated using the comparative 2^ − ΔΔCt method (Ct = threshold cycle) and are reported as fold change (FC) relative to the mean expression of the reference genes.

### miRNA target predictions

The targets for miR-30d-5p were predicted using TargetScan, miRDB and miRTarBase. TargetScan identifies canonical miRNA seed matches and evaluates their evolutionary conservation and context scores. Only conserved sites with a context score < 0.05 were considered significant targets. miRDB uses the MirTarget machine-learning algorithm, trained on experimentally validated miRNA–mRNA pairs to recognize post-transcriptional regulation sequences. Only targets with a confident score > 60 were included. miRTarBase compiles experimentally validated interactions integrating evidence from both low- and high-throughput assays. For reliability, only targets supported by more than one validation method or one publication were selected. Targets were selected based on their overlap among the three databases to ensure reliability and methodological consistency.

### Statistical analysis

Statistical analyses were conducted using the edgeR Bioconductor package for sequencing data, and GraphPad Prism (v.9.0) and SPSS software (v.20; https://www.ibm.com/products/spss-statistics) for the RT-qPCR analyses. The normality of variables was assessed with the Shapiro–Wilk test. Data was expressed as FC or log2 FC for continuous variables or as percentages for categorical variables. For RT-qPCR validation comparisons between two groups were performed using either Student’s t-test or the Mann–Whitney U test, depending on the data distribution. Graphs were carried out using GraphPad Prism (v.9.0) and VennDiagram packages. The *p*-values for sequencing data were adjusted using Benjamini–Hochberg method. *p* < 0.05 was considered statistically significant.

## Supplementary Information

Supplementary Information.

## Data Availability

The raw small RNA-seq data presented in this study are available in the GEO repository under accession number GSE310639 (https://www.ncbi.nlm.nih.gov/geo/query/acc.cgi?acc=GSE310639).
