# Supplementary material for: RAB27A silencing modulates miR-30d-5p expression and extracellular vesicle non-coding RNA cargo in diabetic podocytes
Source: Sci Rep. 2026 Apr 24;16:19051. doi: 10.1038/s41598-026-49921-1 (PMC13280392; doi:10.1038/s41598-026-49921-1)
Supplement: Supplementary file 1 — Supplementary Information. [file 41598_2026_49921_MOESM1_ESM.docx]

*Research article*

*RAB27A* silencing modulates miR-30d-5p expression and extracellular vesicle non-coding RNA cargo in diabetic podocytes

Olga Martinez-Arroyo^1$*^, Ana Flores-Chova^1$^, Lesley Escriva^1^, Marta Mendez-Debaets^1^, Sergio Martinez-Hervas^2,3,4,5^, Cristina Grange^6^, Benedetta Bussolati^6^, Maria Jose Forner^1,4,7^, Josep Redon^1,8^, Raquel Cortes^1,5‡^, Ana Ortega^1,9‡*^

1 Cardiometabolic and Renal Risk Research Group, Biomedical Research Institute of Hospital Clinico de Valencia INCLIVA, Valencia, Spain

2 Service of Endocrinology and Nutrition, Hospital Clinico Universitario of Valencia, 46010 Valencia, Spain

3 Cardiometabolic Risk and Diabetes group, Biomedical Research Institute of Hospital Clinico de Valencia INCLIVA, Valencia, Spain

4 Department of Medicine, Faculty of Medicine, University of Valencia, 46010 Valencia, Spain.

5 CIBERDEM (CIBER of Diabetes and Associated Metabolic Diseases), 28029, Madrid, Spain.

6 Department of Medical Sciences, University of Turin, 10126 Turin, Italy.

7 Internal Medicine Unit, Hospital Clinico Universitario, 46010 Valencia, Spain.

8 CIBEROBN (CIBER of pathophysiology of Obesity and Nutrition), 28029, Madrid, Spain

9 CIBERCV (CIBER of Cardiovascular diseases), 28029, Madrid, Spain

$ These authors contributed equally and share first authorship

‡ These authors contributed equally as senior authors to this work

* Correspondence: *Olga Martinez (omartinez@incliva.es); Ana Ortega* *(aortega@incliva.es)*

**SUPPLEMENTARY MATERIAL**


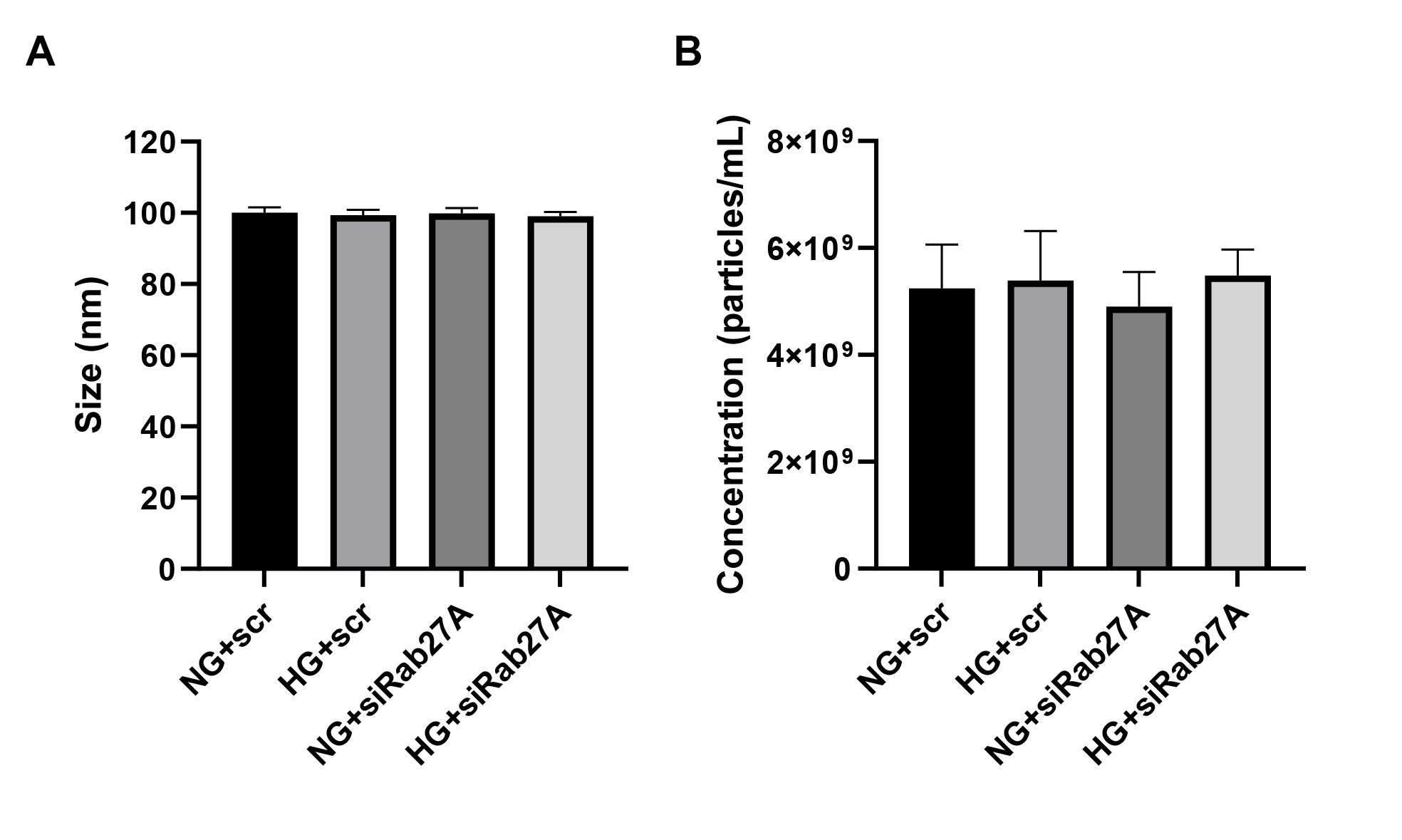


**Figure S1. Size and particle concentration in the different experimental conditions. A** Size (nm) of EVs isolated from glucose treated and/or silenced podocytes. **B.** Concentration, expressed in particles/mL in EV samples from glucose treated and/or silenced podocytes. CNT scr, Control scramble; siRab27A, Rab27A silenced. Data are shown as mean ± SEM (standard error of the mean). N = 5 biological replicates for each group.


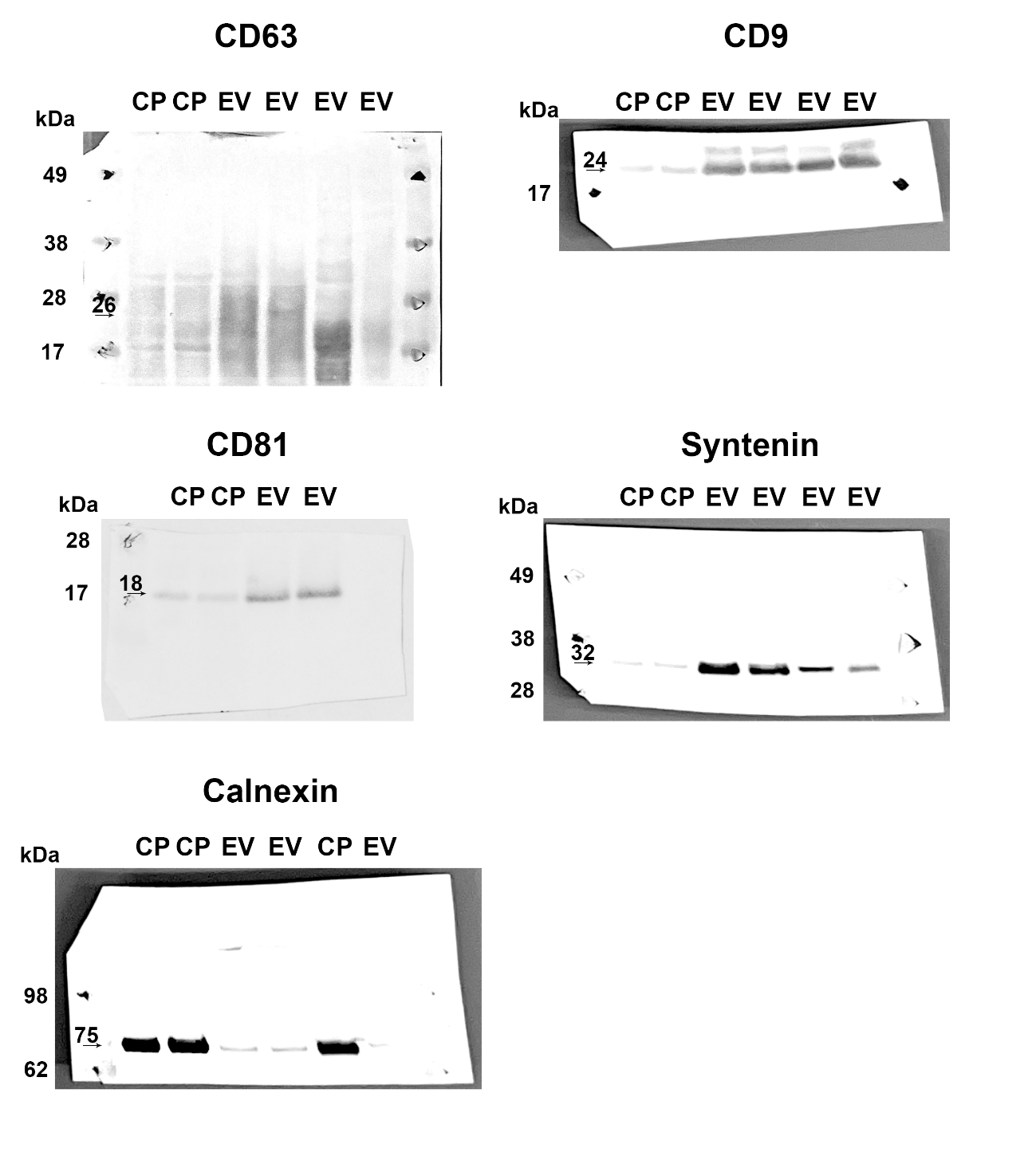


**Figure S2.** **Whole western blot membranes corresponding to Figure 1.** Complete membranes showing EV markers CD63, CD9, CD81, and syntenin, as well as calnexin, an endoplasmic reticulum marker, in podocyte cell pellet (CP) samples and extracellular vesicles (EV) derived from podocyte cultures. Numbers on the left indicate molecular weight markers (expressed in kDa), while arrows, together with the numbers shown above, indicate the molecular weight of the protein of interest.

**
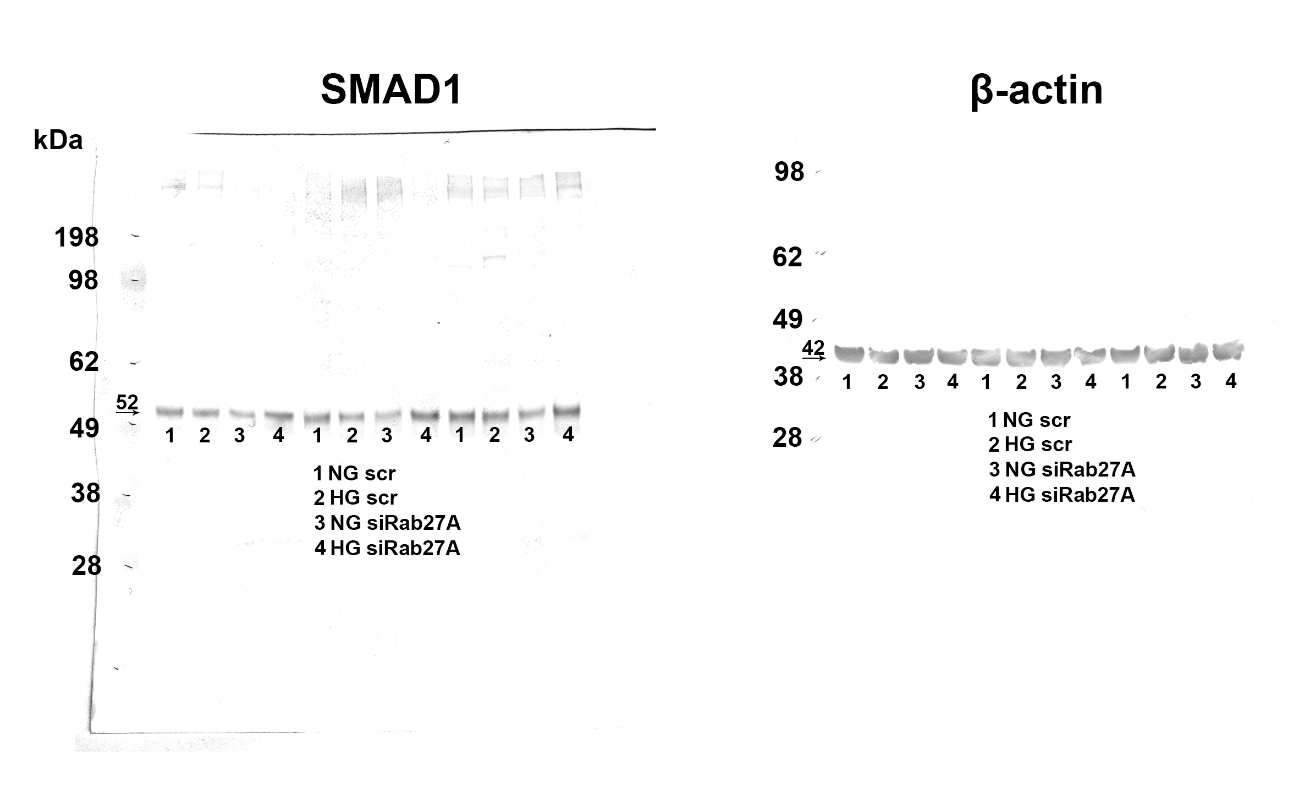
Figure S3.** **Whole western blot membranes corresponding to Figure 4.** Complete membranes showing SMAD1 and β-actin proteins in podocyte samples. Numbers on the left indicate molecular weight markers (expressed in kDa), while arrows, together with the numbers shown above, indicate the molecular weight of the protein of interest.


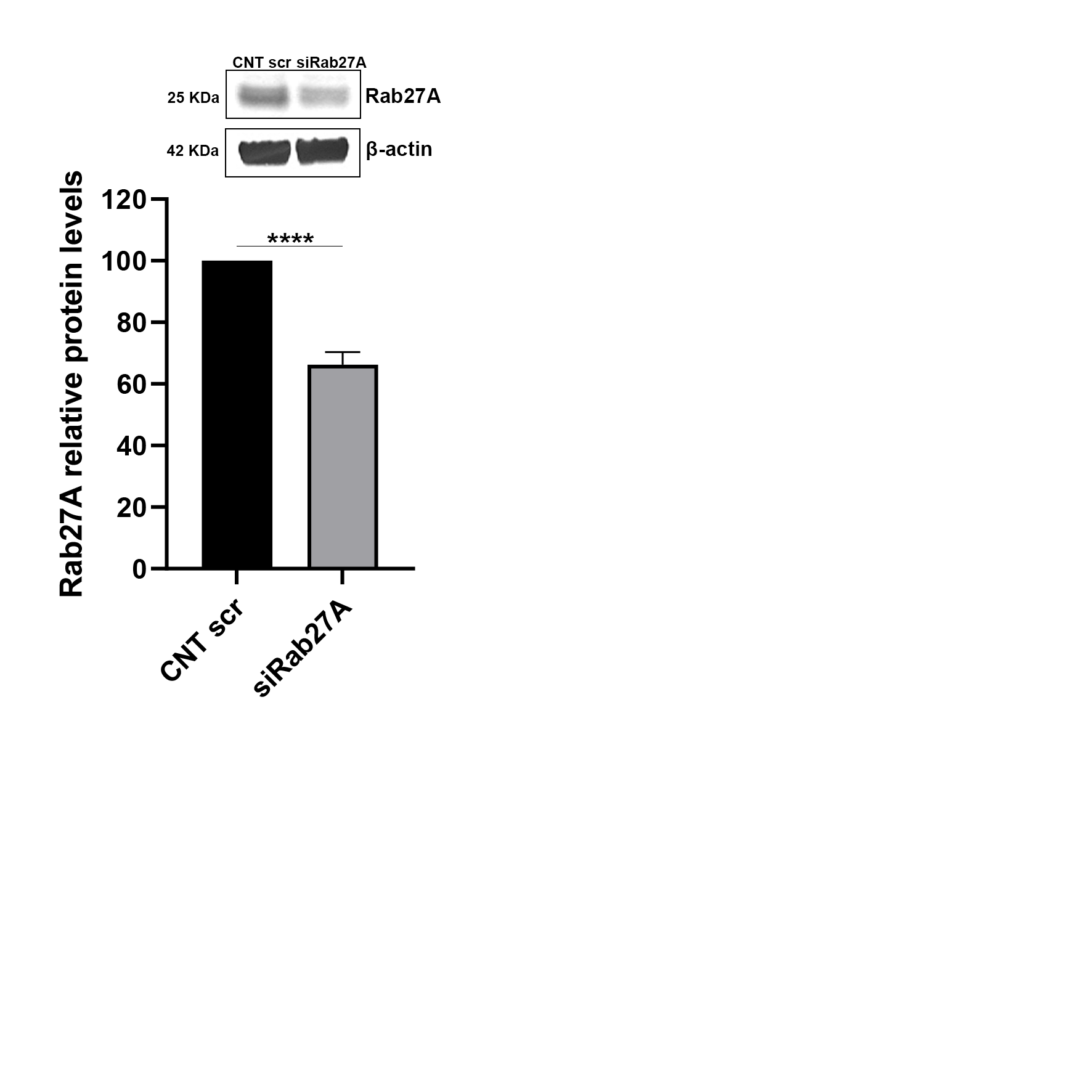


**Figure S4.** **Protein levels of Rab27A** **in siRab27A transfected podocytes.** Western blot membranes and graph showing protein levels of Rab27A and β-actin (loading control). CNT scr, Control scramble; siRab27A, Rab27A silenced. Data are shown as mean ± SEM (standard error of the mean) and protein levels are normalised to 100. N = 5 biological replicates for each group. Statistical significance is indicated as ****p < 0.0001.
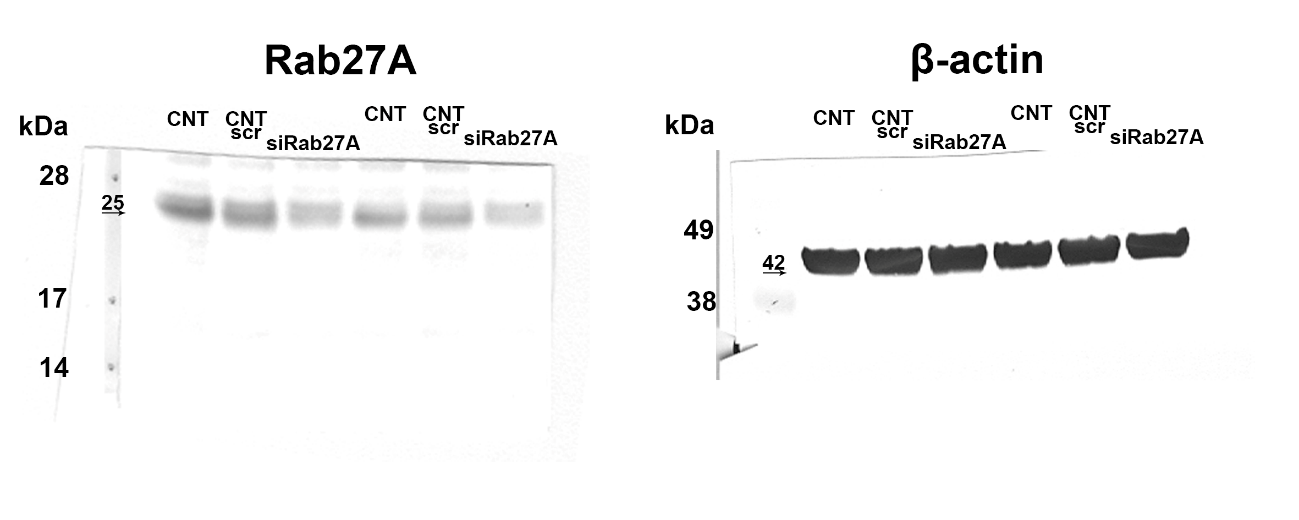


**Figure S5.** **Whole western blot membranes corresponding to Figure S4.** Complete membranes showing Rab27A and β-actin proteins in podocyte cell pellets from control (CNT), control scrambled (CNT scr) and Rab27A silenced (siRab27A) samples. Numbers on the left indicate molecular weight markers (expressed in kDa), while arrows, together with the numbers shown above, indicate the molecular weight of the protein of interest.
